# Water Heater Type, Temperature Setting, Operational Conditions, and Insulation Affect Ecological Niches for *Legionella* Growth

**DOI:** 10.1021/acsestwater.4c00894

**Published:** 2024-12-23

**Authors:** Fernando A. Roman, Rebekah L. Martin, William J. Rhoads, Annie Pearce, Rania E. Smeltz, Amy Pruden, Marc A. Edwards

**Affiliations:** † Department of Civil and Environmental Engineering, Virginia Tech, Blacksburg, Virginia 24061, United States; ‡ Department of Civil and Environmental Engineering, Virginia Military Institute, Lexington, Virginia 24450-0304, United States; § Black and Veatch Corporation, Overland Park, Kansas 66211, United States; ∥ Department of Building Construction, Virginia Tech, Blacksburg, Virginia 24061, United States

**Keywords:** premise plumbing, insulation, temperature, drinking water, *Legionella*

## Abstract

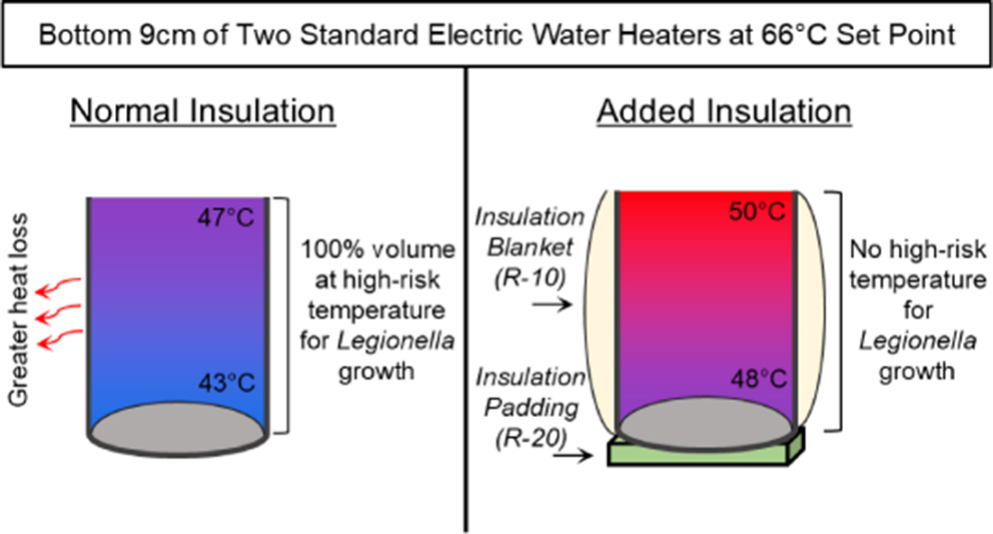

Residential water heating represents an important nexus of energy/water conservation, waterborne disease, hygiene, and consumer preference. Here, we examine attributes of two off-the-shelf 151-L tank water heaters, one with hot water recirculation (recirculating) and another without recirculation (standard), compared to a tankless on-demand heater (on-demand). Energy efficiency decreased in the order on-demand > standard > continuous recirculation. However, the electric on-demand water heater repeatedly malfunctioned and could not consistently achieve target temperatures >48 °C. At a temperature setting of 48 °C, the volume of water in the pipe and tank within a temperature range at very high risk for *Legionella* growth (38–47 °C) decreased from recirculating (150 L) > standard (40 L) > on-demand (∼0.47 L). However, at a temperature setting of 66 °C, the standard tank was stratified, and the bottom 13 L fell within the very high-risk temperature range, whereas the recirculating tank system maintained 100% of its volume >55 °C, which is not suitable for *Legionella* growth. Addition of insulation was found to markedly increase the temperature throughout the tank. In the standard tank set at 66 °C with insulation, no volume was maintained within the very high-risk range. Insulation can holistically increase energy efficiency and reduce health risks at a sufficiently elevated temperature setting.

## Abstract 2

Through head-to-head laboratory testing, water heater configuration and temperature setting conditions are identified to maximize energy savings while minimizing potential for *Legionella* growth.

## Introduction

Residential water heating in the U.S. accounts for 19% of home energy consumption^1^ at an average annual household cost of $400–600.^2^ The U.S. Department of Energy (DOE) and the U.S. Environmental Protection Agency (EPA) help guide consumer decisions through Energy Star,^3^ whereas the U.S. Green Building Council’s (USGBC) Leadership in Energy and Environmental Design (LEED) rewards both energy and water conservation in their certification criteria.^4−7^ Hot water storage tanks are used in roughly 93% of U.S. single-family homes and are predominantly heated by gas (53%) or electricity (41%).^8^ Conventional tanks can be designed or retrofitted to recirculate the heated water in a loop throughout the building to avoid the need for flushing cold water from the system before showering.^4^ Tankless water heaters, which heat water as needed with minimal storage volume, are only used in approximately 7% of single-family homes.^8^

Each water heater type, design, and plumbing configuration will create distinct water and energy efficiencies.^4^ However, these design and operational aspects also profoundly shape the microbial ecology of the hot water system and the propensity for pathogens to establish and proliferate.^4,9−13^ Pathogens of concern that can establish and grow in potable water distribution systems, especially premise (i.e., building) plumbing, are collectively known as opportunistic premise plumbing pathogens (OPPPs).^14−16^ Some of the key OPPPs of concern for hot water systems include nontuberculous mycobacteria (NTM), *Legionella*, and *Naegleria fowleri*.^15−19^ Among these, *Legionella*, the agent of Legionnaires’ disease, is now the leading cause of reportable waterborne illness in the U.S.^20^

Temperature is a master variable governing the ability of *Legionella* to grow,^21^ with the highest growth rates at water temperatures ranging from 38 to 47 °C (Table 1),^21^ a range that is commonly encountered in tank hot water systems operated at the EPA-recommended set point of <49 °C.^22^ On-demand water heaters are hypothesized to circumvent risks posed by tank water heaters because the storage volume and surface area available for microbial growth are drastically reduced.^22^

**Table 1:** *Legionella* Growth Potential across Various Water Temperature Ranges Adapted from the National Academies of Science, Engineering, and Medicine *Legionella* Report^21^

Water Temperature Range (°C)	*Legionella* Growth Potential
<25	None
25–32	Low
32–38	Moderate
38–47	Very High
47–50	Moderate
50–55	Very Low
>55	None

Without recirculating pumps to provide mixing, standard electric water heaters thermally stratify, resulting in a layer of dense cool water and sediment below the lowest tank heating element and creating temperatures suitable for *Legionella*(23) and *L. pneumophila* growth.^22^ Electric water heaters with recirculating pumps, or gas water heaters that heat from the bottom of the tank, are not prone to stratification.^22^

Standby energy and heat losses in tank-based systems can be reduced by lowering the system temperature, minimizing pipe lengths, or adding insulation to the pipes and tanks.^2,24^ There are obvious trade-offs between set point temperature and the tank volume that is at the optimal temperature for *Legionella* growth, particularly for recirculating tanks.^22^ Specifically, if tank set points are lowered to save cost and energy, they may continuously recirculate temperatures adequate for *Legionella* growth, even without the stratification issues experienced in standard water heaters.^22^ In contrast, a tankless system set at a high temperature (>55 °C) contains very hot water when in use and quickly drops to room temperature water when it is not, with temperatures only transiently existing in the range for ideal *Legionella* growth.^22^

Here, we examine the effects of water heater system type, plumbing configuration, and temperature settings on energy demands and the extent to which optimal temperatures for *Legionella* growth are maintained. Specifically, we compare two tank systems, one nonrecirculating (standard) and one with a recirculating plumbing configuration (recirculating), and a tankless (on-demand) system configuration using off-the-shelf residential-scale system components. Phase 1 of the study examines energy efficiency, delivered water temperature, and the volume of water at risk for *Legionella* growth by scaling up a prior study using small apartment-sized 75.7-L (20-gallon) capacity heaters^25^ to standard 151-L (40-gallon) tank heaters with 15.2 m of cross-linked polyethylene (PEX) pipe to the point of use (POU). In phase 2 of the study, we examine the impact of the external insulation and temperature set point on the volume of water maintained within the optimal growth range for *Legionella* in a standard heater with a standard plumbing configuration. The overall goal was to better understand the key factors that impact water and energy conservation for each configuration and how these factors affect habitat in a temperature range suitable for *Legionella* growth in water delivered to consumers.

## Materials and Methods

### Phase 1: Energy and Temperature Implications of Water Heater Configuration

#### System Design

**Figure 1:**
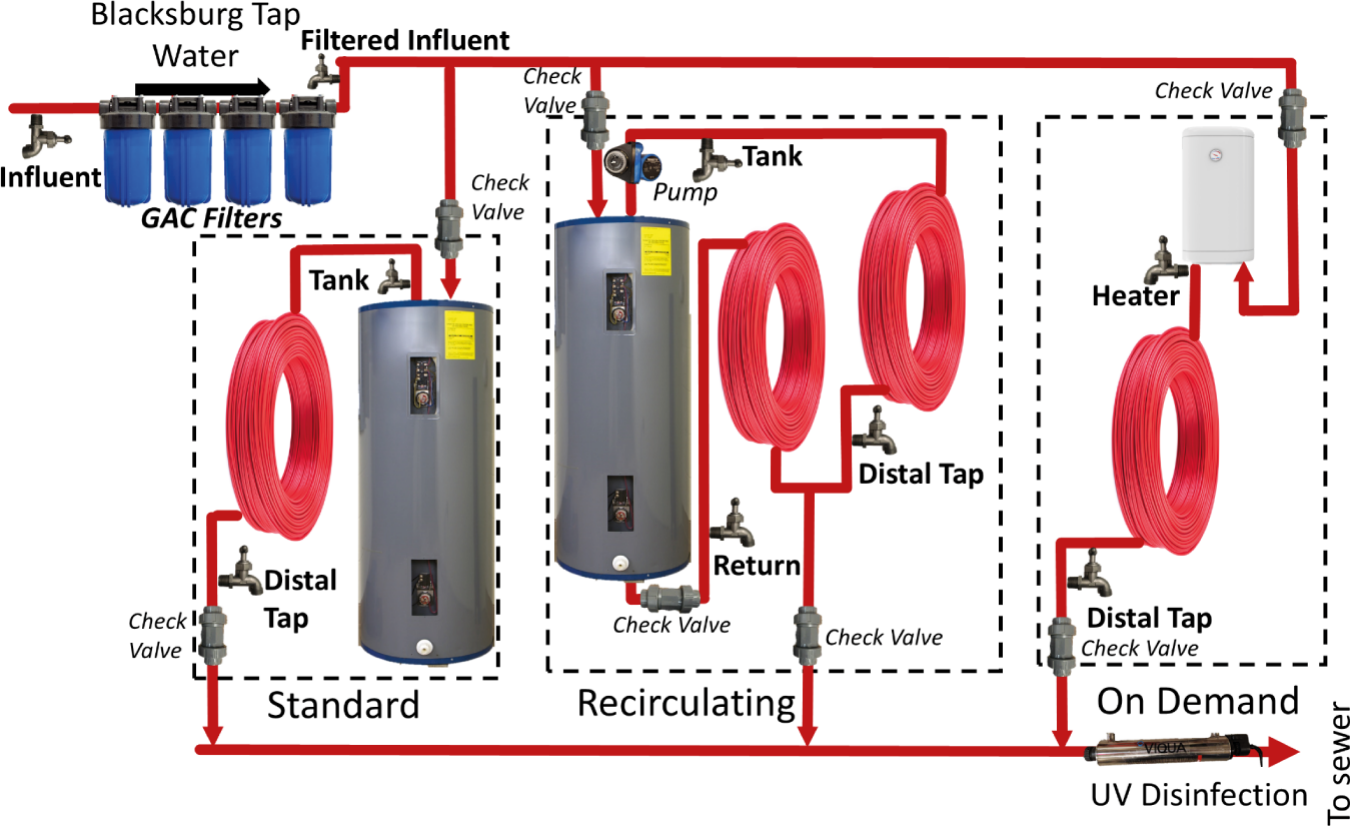
Parallel comparison of water heater types and plumbing configurations in phase 1. Three systems were designed with check valves at the entrance and exit points of each system. Two or three sampling ports were included in each system and are labeled as tank*/*heater, distal, or return. Blacksburg tap water was used as the influent for the system after being filtered through multiple GAC filters.

Independent standard, recirculating, and on-demand hot water systems were plumbed with electric water heaters (Figure 1). The standard system was equipped with a glass-lined 151 L (40 g) steel storage tank (model XE40M06ST45U0, Rheem, Atlanta, Georgia, United States) and 15.2 m of 19 mm (inner diameter) PEX pipe to simulate typical plumbing within a residence serving a distal sink or shower (Figure 1). Two PVC sampling taps were incorporated into the system just after the hot water outlet (tank) and at the end of the 15.2 m of PEX pipe (distal). The recirculating system consisted of identical materials and components but with the addition of a hot water recirculation pump (500800 Premier, Watts, North Andover, Massachusetts, United States) run continuously with an extra 15.2 m PEX water return loop, and a third sampling point at the end of the return loop (return) (Figure 1). Tank systems were equipped with flow meters (model F-400, Blue-White Industries, Huntington Beach, CA, United States) and temperature profile sensors (HOBO U12, ONSET, Bourne, Massachusetts, United States) throughout the depth of the water heater. All the plumbing in each closed system used cross-linked polyethylene (PEX) (type b) with plastic connectors, with four to six brass connectors or adapters for each tank system.

The on-demand system consisted of a copper 240 V heater (AE9.5 PowerStar, Bosch Water Heating, Waitsfield, Vermont, United States) plumbed with a 15.2 m length of 19 mm (inner diameter) PEX pipe (Figure 1). A PVC sampling port was included just after the instantaneous heater (heater) and at the end of the 15.2 m of PEX pipe (distal).

#### System Operation

Phase 1 of testing used two representative set point temperatures (40 and 48 °C), considered to be near the very high *Legionella* growth potential range (38–47 °C) (Table 1),^21^ as well as the optimal growth range (32–42 °C) for *L. pneumophila*.^13^ Flushing of the system took place at a regular 8 h interval (3× per day). Flush volumes were either 37.8, 75.7, or 151 L (10, 20, or 40 gallons) per event (i.e., 0.75, 1.5, or 3 tank volumes turnover per day), representing a span of low, medium, and high flush volumes. These flush volumes roughly correspond to low-, moderate-, and high-range consumer water demands, as defined by Brazeau and Edwards (henceforth defined as low, medium, and high).^25^ The recirculating and standard systems were both flushed at 3.78 L/min (1 gal/min), whereas the on-demand flow rate sometimes had to be lowered to 1.89–3.78 L/min (0.5–1 gal/min) in order to achieve temperature targets. The three water heaters were first tested using the low flush rates, followed by the medium and then high flush rates. For each flush rate, the three water heaters were first tested at 40 °C, followed by 48 °C (see Table S1).

#### Inoculation with *L. pneumophila*

During the first 4 years of system operation, a multitude of unsuccessful attempts were made to establish *L. pneumophila* by inoculation. Research herein was conducted during this phase of the work.

#### Sampling

Delivered temperature in the standard and recirculating heaters was measured in effluent continuously flowing at 3.8 L/min through a 500 mL beaker by using wireless data logger temperature probes (HOBO model U12). The data logger took measurements every 10 s until the end of the flush period. The on-demand configuration was tested using a manual thermometer (H-B Durac Plus Thermometer, SP Scienceware, Warminster, Pennsylvania, USA) due to physical location constraints with the POU in the designated laboratory space. Measurements were taken every 15 L at a flow rate of 2 L/min.

Internal tank temperature measurements were taken using a sampling rod apparatus that was used to temporarily replace the tank anode rod (Figure 1). Wireless data logger temperature probes (HOBO model U12) were attached to the sampling rod and collected measurements every 10 s while the system was pressurized and operational. These data were collected over a single flushing period (8 h) when the water heater was set at 48 °C and the flush time was 40 min.

Energy measurements were taken using electric watt meters (Elite 4.0, Efergy, Sheffield, England, United Kingdom) that were placed along the power supply for each of the heaters and measured AC current, which were aggregated into cumulative kWh values. Measurements were recorded over the course of one flush, and the daily demand was noted weekly as a backup to the data storage in the monitors throughout phase 1. A separate monitor (P4400 Kill-A-Watt Electricity Monitor, P3 International, New York, New York, United States) determined energy use by the recirculating pump (recirculating system only). Energy efficiency calculations were performed as described by Brazeau and Edwards, where energy efficiency is defined as the increased energy in hot water delivered to the tap, divided by total energy consumed.^24^ Details of the seasons sampled, influent temperature, and the number of samples corresponding to each flush rate and configuration’s set temperature are provided (Table S1).

### Phase 2: Insulation Tests with Standard Water Heater Configuration Set at 66 °C

The standard configuration system from phase 1 was later utilized to test the effects of external tank insulation on the temperature profiles (Table 2). The top and bottom heating elements of the heater were set to their maximum set points of 66 °C and allowed to run for 45 h to allow the temperature profile in the tank to stabilize (Figure S2). A 139 cm temperature probe (PRB-K-135, ThermoWorks, American Fork, Utah, United States) with an accuracy of ±0.5 °C was then lowered from the top of the water heater to get a vertical temperature profile of the tank in increments of 7.6 cm for each configuration to act as a control for a standard water heater without any added insulation (Table 2). This process was then repeated on the same water heater for a variety of insulation conditions and with both heating elements either set at 66 °C or with the bottom heating element either disconnected or at a set point of 52 °C (Table 2). For additional comparison, the recirculating tank from phase 2 was allowed to run continuously for 45 h without the use of added insulation.

**Table 2:** Insulation Configurations Tested during Phase 2 for a Conventional Tank Water Heater with Standard Plumbing Configuration^a^^b^^c^

	Temperature Set Point (°C)		
Insulation Configuration	Top Heating Element	Bottom Heating Element	Purpose
No added insulation	66	66	Determine the vertical temperature profile without any added insulation (control)
*R*-10 blanket	66	66	Determine the effect of an insulation blanket^ on the temperature profile
*R*-20 padding	66	66	Determine the effect of an insulation padding# placed beneath the water heater on the temperature profile
*R*-10 Blanket and *R*-20 padding	66	66	Determine the effect of an insulation blanket + insulation padding placed beneath the water heater on the temperature profile
*R*-10 Blanket and *R*-20 padding	66	52	Determine the effect of a lower bottom heating element set point on the temperature profile in a well-insulated water heater
*R*-10 Blanket and *R*-20 padding	66	--*	Determine the effect of a faulty bottom heating element on the temperature profile in a well-insulated water heater

a *Indicates a disconnected heating element.

b ^Insulation blanket consisted of a 7.62 cm (thickness) × 121.9 cm (height) × 190 cm (width), off-the-shelf (Frost king Vinyl Faced Fiberglass, Thermwell Products Co. Inc., Mahwah, New Jersey, United States) water heater insulation blanket (*R* rating of 10).

c #Insulation padding placed beneath the water heater consisted of four 25.4 cm insulation boards (GreenGuard Type IV, Kingspan, Kingscourt, Ireland) with an *R* rating of 5 per 25.4 cm (for a total *R* rating of 20 with 4 of them stacked below the tank).

#### Statistical Analyses

R version 3.4.3 was used for statistical analyses. Data were tested for normality using a Shapiro −Wilk test. The Kruskal–Wallis rank sum test was used to determine significant differences between groups of data, specifically comparing energy efficiency with influent temperature, season, flush volumes, and water heater set point. Statistical significance was set at *p* < 0.05.

## Results

### Phase 1: Energy and Temperature Implications of Water Heater Configuration

#### Energy Efficiency

**Figure 2:**
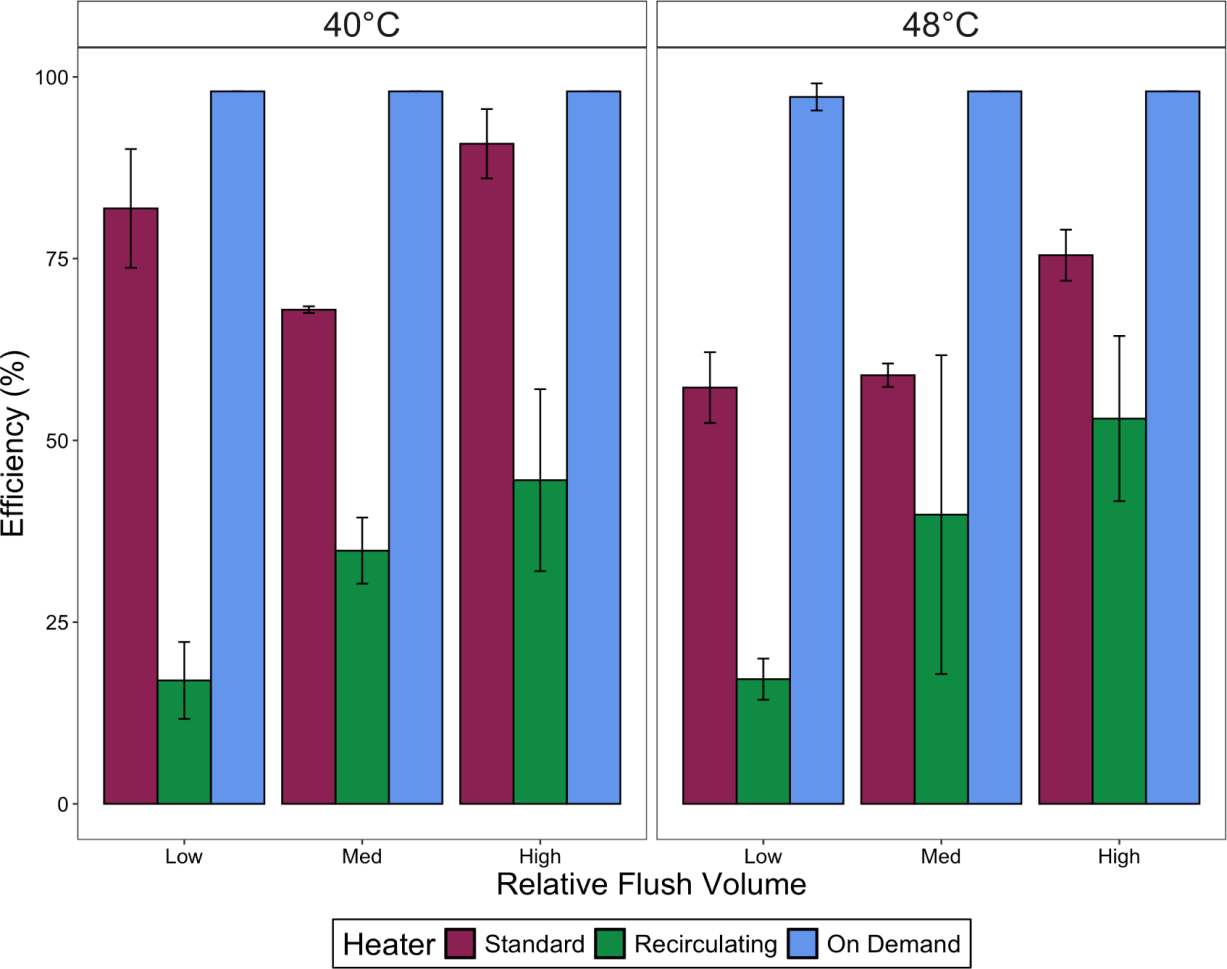
Energy efficiency of recirculating, standard, and on-demand heater configurations vs flush volume. Low flush volume was 37.8 L (10 gallons), the medium flush volume was 75.7 L (20 gallons), and the high flush volume was 151 L (40 gallons). Each system was flushed 3× per day with 8 h of stagnation between flushes. Error bars are 95% confidence intervals for *n* = 3–5 measurements for each condition.

The recirculating configuration resulted in the lowest energy efficiency of all three configurations (Figure 2). Increasing the flush volume generally acted to increase the percentage efficiency of the recirculating configuration, with ∼17% efficiency at low flush volumes and a significant increase up to ∼50% efficiency at high flush volumes (Kruskal–Wallis rank sum test, *p* = 0.0002). Decreasing the temperature from 48° to 40 °C increased the efficiency of the recirculating system by only 1–9% (Kruskal–Wallis rank sum test, *p* = 0.62). The standard tank heating efficiency was much higher than the recirculating system, at 68–90% when set at 40 °C and 57–75% when set at 48 °C (Kruskal–Wallis rank sum test, *p* = 0.005). When combining the 40 and 48 °C data sets, the standard system also differed between low, medium, and high demand scenarios (Kruskal–Wallis rank sum test, *p* = 0.02) (as shown in Figure 2). The on-demand system was virtually 100% efficient in all situations, as it operated with a minor storage volume and only used energy when the flush was occurring, resulting in nearly complete conversion of input energy to deliver hot water and little heat loss to the environment.

#### Delivered Water Temperatures and Implications for Consumer Comfort and Cost

**Figure 3:**
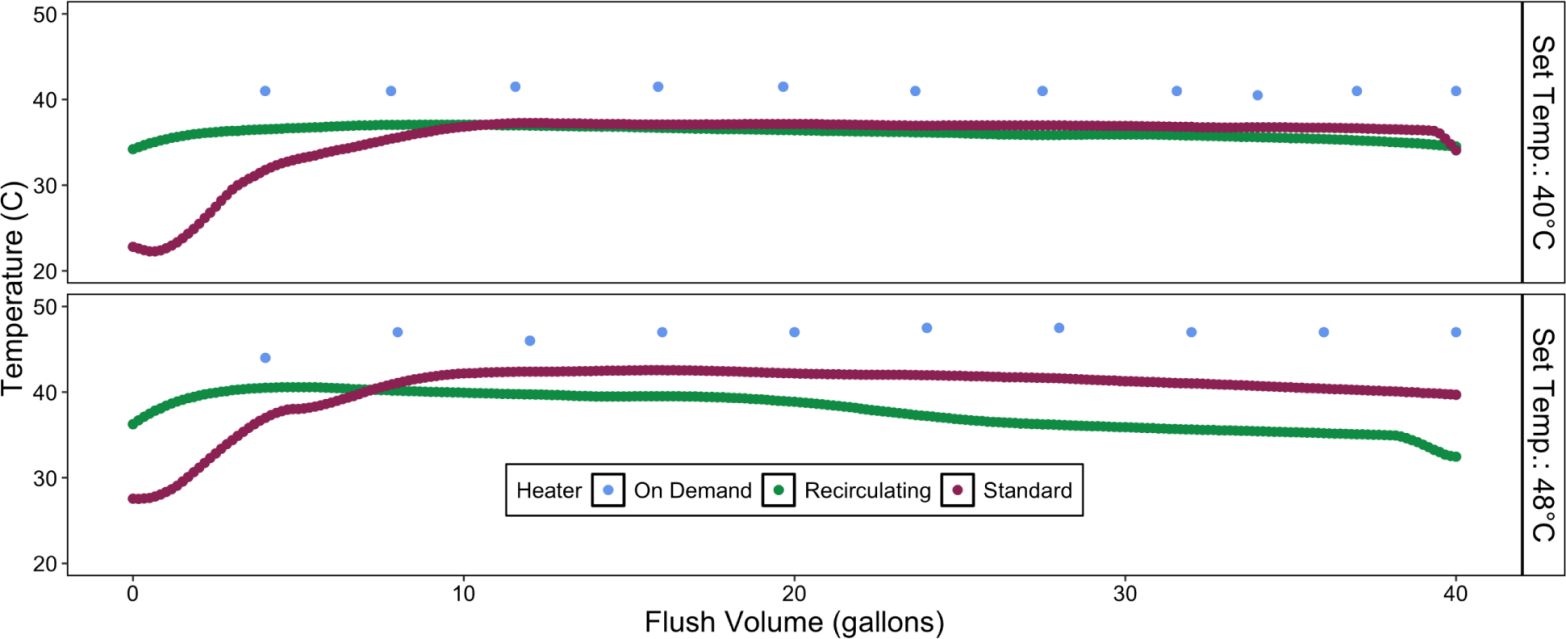
Trends in delivered water temperature at 40 and 48 °C water heater set points vs flush volume from each water heater configuration. Recirculating and standard heaters were sampled using temperature logging probes. The on-demand heater water temperature was measured manually with a thermometer.

Consistent with the everyday experience of consumers and corresponding wait times to achieve temperatures suitable for showering, the standard system required flushing of 30.2–37.8 L (8–10 gallons) before water at the maximum temperature emerged from the tap (Figure 3). The recirculating system set at 48 °C reached a maximum temperature faster but also did not effectively maintain a stable elevated temperature to the degree that the standard system achieved. This was due to the continuous mixing of the influent cold water entering the tank as water is withdrawn from the system. The on-demand system continuously delivered warm water at the set point throughout the entire flush period and theoretically could do so indefinitely (Figure 3). However, due to the extremely high energy requirements to instantly heat water, the on-demand system could not deliver water at the desired flow of 3.78 L/min (1 gal/min) and had to be reduced to 1.89–3.78 L/min (0.5 gal/min) to achieve a target temperature of 48 °C. A flow rate of 1.89 L/min (0.5 gal/min) was also the minimum required for the tankless water heater to operate. Thus, there are inherent strengths and limitations of each electric heater type and configuration examined in this study in terms of the consumer experience, including the wait times for hot water, the maximum temperature achieved, achievable flow rates, and the duration that hot water is maintained in a comfortable range for bathing purposes.^24^

#### Estimating System Volumes Maintained within the Temperature Range at Risk for Legionella Growth

**Figure 4:**
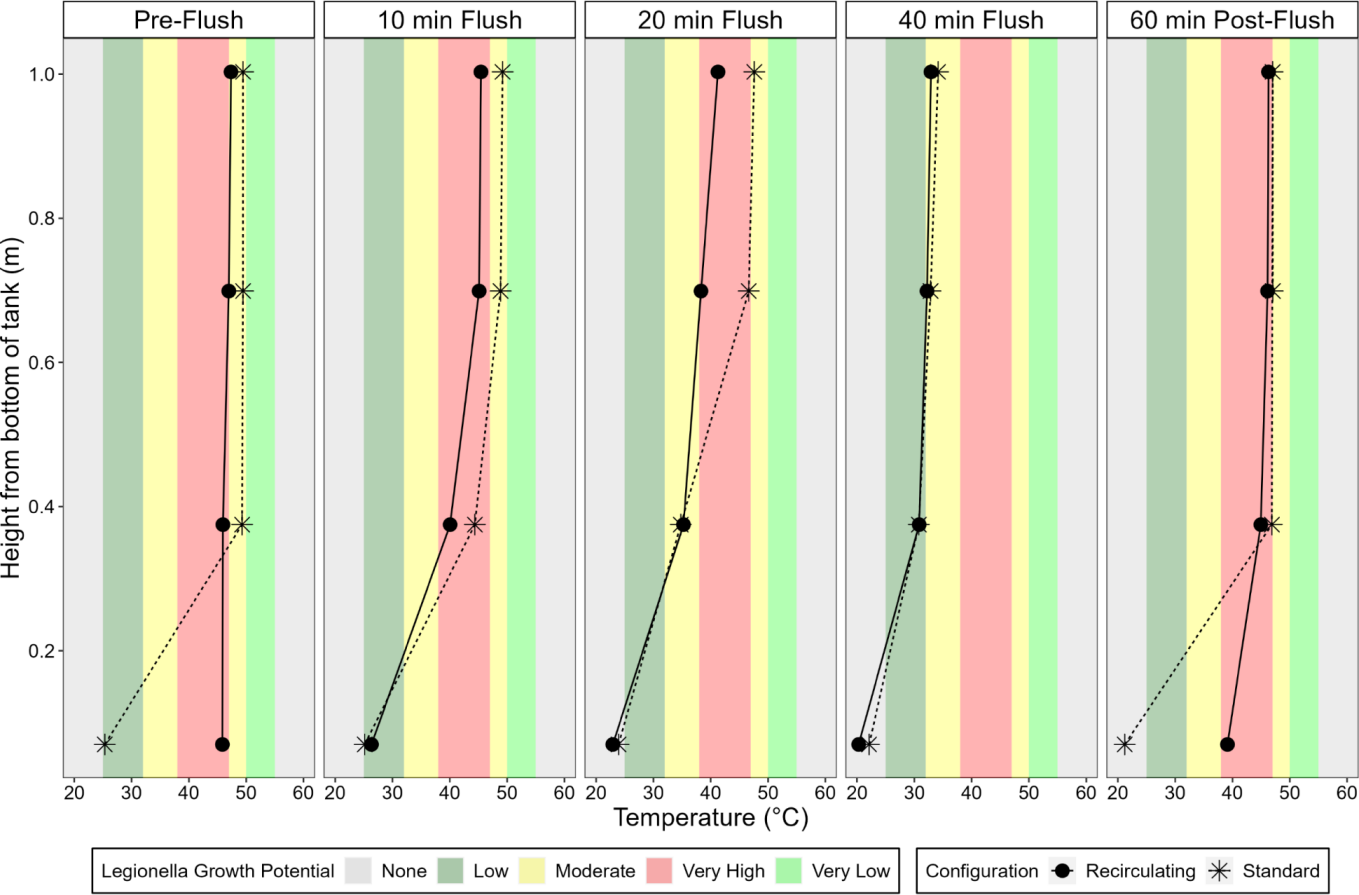
Internal temperature profile in the recirculating and standard water heater tank before, during, and after a representative 40 min flush event. Both heater configurations were set at a temperature of 48 °C. *Legionella* growth potentials are defined as indicated in Table 1.

The internal temperatures maintained in the water heater tank between water use events were measured and compared to reported temperature niches for *Legionella* growth (Figure 4). For example, due to stratification in the standard tank, the temperature was cooler than the *Legionella* “low” growth threshold at the bottom (∼0 m) of the tank at all times, even when set at 48 °C and after 8 h heating. However, for the recirculating configuration set at 48 °C, the bottom of the tank was maintained at the “very high” growth temperature range for 7^+^ hours, only dropping in temperature temporarily during active use (Figure 4). At a depth of 0.375 m above the bottom of the tank, the temperature in the standard tank was higher than in the recirculating tank (49 °C compared to 46 °C) and trended into the moderate growth zone for periods of 1 to 8 h during recovery. On the other hand, the recirculating tank temperature was always maintained in the “very high” zone for growth 0 to 0.7 m above the bottom of the tank, from 60 min (post-flush) until the next flush cycle (pre-flush) for a total span of 7 h when set at 48 °C (Figure 4). Thus, at 48 °C, the majority of the volume of the recirculating tank was consistently at “very high” risk, whereas the standard tank was almost always at low or moderate risk temperature (Figure 4). Integrating over an entire flush cycle, including 8 h stagnation time, indicates that 26% of the standard tank configuration (tank + pipes) volume was in the very high-risk range (by time-weighted volume), whereas 94% (∼150 L) of the recirculating tank configuration (tank + pipes) volume was at very high risk for *Legionella*.

The volume of water maintained within the very high-risk temperature range for *Legionella* was minimal for the on-demand system, with only 10% (∼0.47 L) of the system’s volume (on-demand heater + pipes) at risk over a flush and stagnation cycle. The on-demand system also stored only a small fraction of water capacity in the heating device relative to the tanks (0.07% volume; Table S2).

### Phase 2: Factors Affecting Vertical Temperature Profiles for Water Heater Set at 66 °C

#### Comparison of Different Insulation Conditions with Both Heating Elements Set at 66 °C

**Figure 5:**
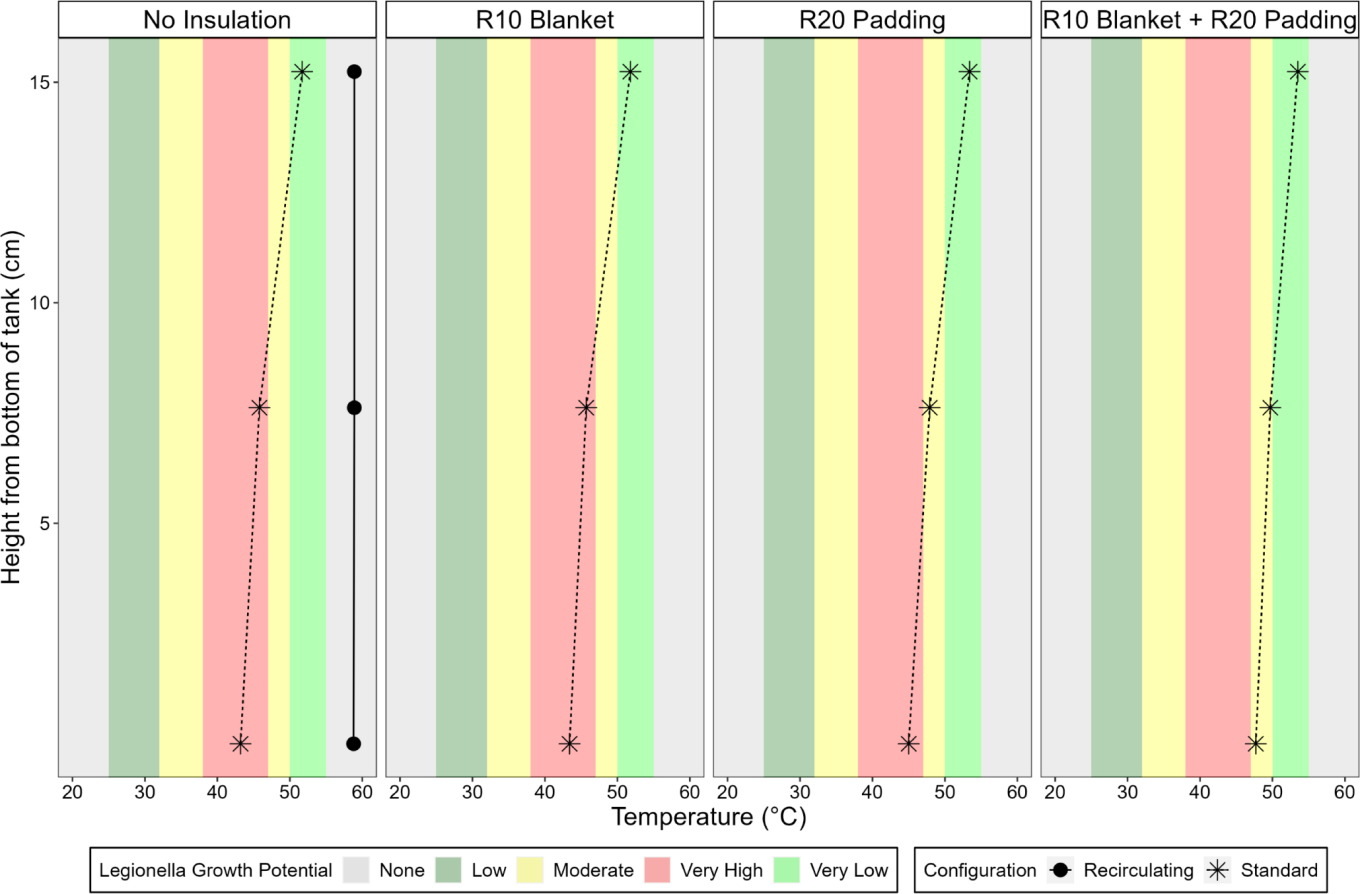
Internal temperature profile of the conventional tank water heater with standard configuration, testing different insulation conditions (Table 2), compared to a recirculation configuration with no added insulation with both heating elements set to 66 °C. Systems were subject to stable operation at each indicated condition for 46 h prior to testing. Measurements are shown for a representative operational run. *Legionella* growth potentials are defined as indicated in Table 1.

When the lower and upper heating elements were set to their maximum set point of 66 °C, the temperature at the very bottom surface of the standard tank was still only 43.2 °C. The addition of an *R*-10 insulation blanket had a negligible effect on this temperature (43.4 °C). The temperature with and without the insulation blanket was also virtually identical at 7.6 cm above the bottom (45.7 vs 45.8 °C) and 15.2 cm above the bottom (51.7 vs 51.8 °C) of the tank. Thus, at a minimum, the volume corresponding to the bottom 7.6 cm depth was always maintained at a very high-risk temperature for *Legionella* growth using the standard tank and typical blanket insulation. Using a linear interpolation of temperature between 7.6 and 15.2 cm, it is estimated that the true tank depth maintained in the very high-risk temperature range for *Legionella* likely extended to ∼9.2 cm (∼13 L of the total volume) above the bottom of the tank (Figure 5).

When the standard tank was set on top of an *R*-20 insulation pad, the water was hotter at all depths below the tank’s bottom heating element. For instance, with just *R*-20 padding and no additional tank insulation elsewhere, the temperature was 45 °C at the tank’s bottom surface, 47.9 °C at 7.6 cm above the bottom of the tank, and 53.4 °C at 15.2 cm above the bottom of the tank. While the bottom surface temperature of the *R*-20 insulation pad condition maintained a temperature still considered very high risk for *Legionella*, assuming a linear interpolation, the depth at very high risk extended to only ∼5.2 cm above the bottom of the tank (Figure 5). If the water heater on the *R*-20 pad was additionally wrapped with the *R*-10 blanket, the temperature was 47.7 °C at the bottom, 49.7 °C at 7.6 cm above the bottom of the tank, and 53.5 °C at 15.2 cm above the bottom surface. Hence, the use of both blanket and insulating floor pads effectively eliminated the volume at very high risk for *Legionella* growth (Figure 5). By comparison, for the condition where a water heater of the same model was fitted with a recirculation system, without any added insulation, temperatures of 58.8–58.9 °C were found throughout all depths of the completely mixed tank, also indicating that no portions of the tank were at risk for *Legionella* growth (Figure 5).

#### Effect of Lower Set Points and Inoperable Lower Heater Element

**Figure 6:**
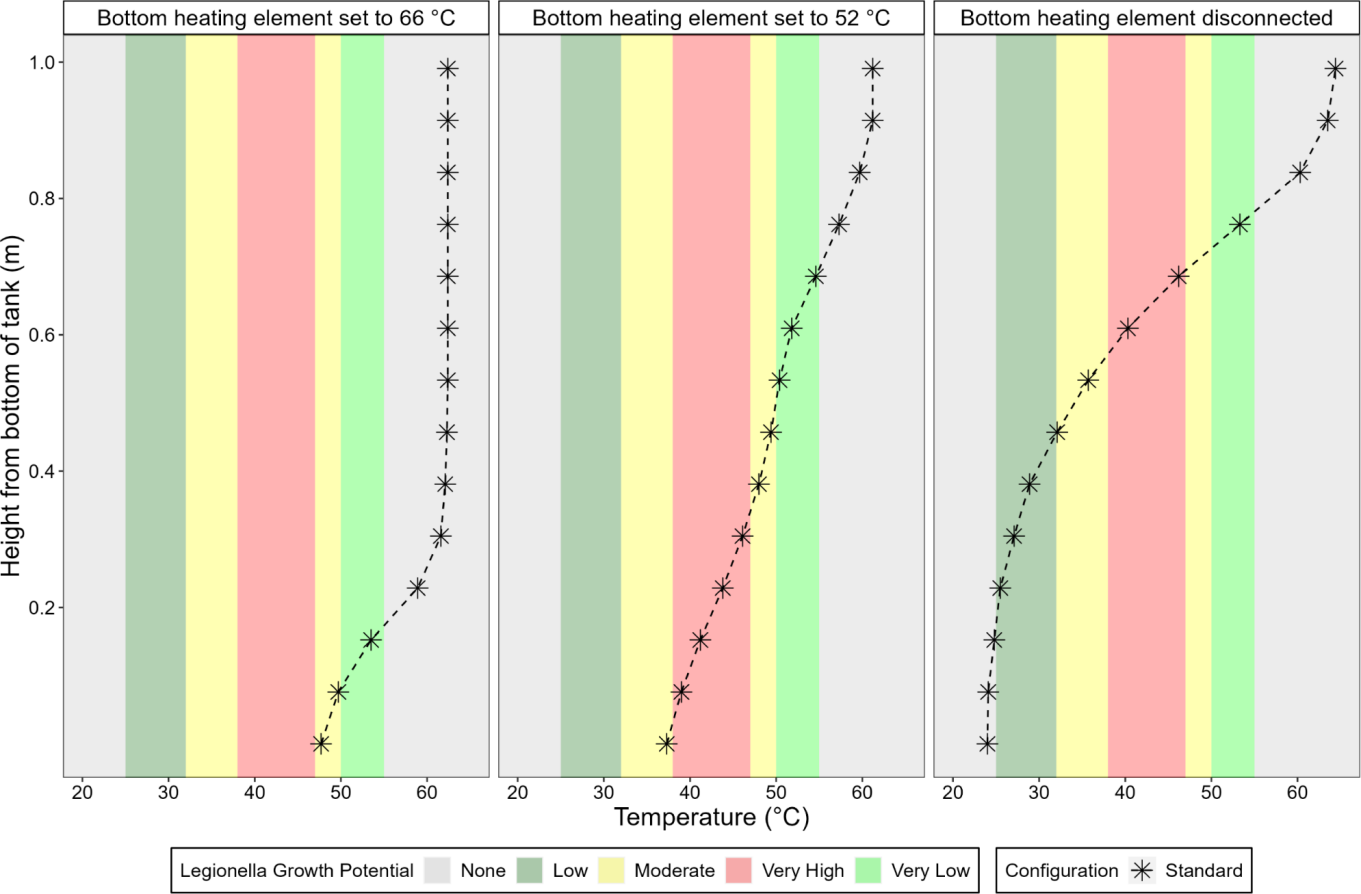
Internal temperature profile of the conventional tank water heater with standard configuration as a function of different bottom heating element set points, with the top heating element consistently set to 66 °C. The tank was equipped with optimal insulation for this experiment, i.e., an *R*-10 water heater insulation blanket and *R*-20 insulation pad. Systems were subject to stable operation for 46 h at each new setting prior to testing. Measurements are shown for a representative operational run. *Legionella* growth potentials are indicated as defined in Table 1.

Consumers can independently set the bottom or top thermostats to a different temperature. If the bottom heating element was set to 52 °C while the top element was set to 66 °C, the entire depth up to 30.5 cm above the bottom of the water heater fell into a temperature range considered at very high risk for *Legionella* growth (Figure 6). When the bottom heating element was turned off, to represent a relatively common situation of a burned-out element in hard waters,^26^ depths up to 61 and 68 cm above the bottom of the water heater were held at temperatures considered at very high risk for *Legionella* growth. However, the lower regions of the tank at and beneath 15.2 cm depth then became cold enough (24–24.8 °C) to inhibit growth of *Legionella* (Figure 6).

## Discussion

### Energy Efficiency and Temperature of the Delivered Water

Similar to previous studies in our laboratory using smaller apartment-sized water heaters,^22,24,25^ the full-size 151-L (40-gallon) water heaters with standard configuration and insulated pipes outperformed the recirculating configuration in terms of energy efficiency. As in the case of Brazeau and Edwards,^25^ the recirculating system was also not as effective as a standard system in providing stable hot water temperatures at the tap, due to mixing of incoming cold water (Figure 3). Delivered water temperature is another important aspect of system performance. Specifically, even when the water heaters are set at the same temperature, the water flowing to the distal POU varies distinctly with time for a standard versus recirculating system in a manner that can be perceived by consumers and thus likely affects use patterns. One approach to increasing the energy efficiency of the recirculating configuration would be to optimize the pump operation; e.g., setting the pump so that it only switches on at peak use times could reduce the pump runtime to as low as 14–25% of the day and likely increase energy efficiency.^24,27^ This would also expectedly reduce energy losses of the recirculating system.

The on-demand system performed according to expectations with near-perfect energy efficiency,^5,25^ but the unit could not reliably deliver 3.78 L/min (1 gallon/min). The on-demand water heater itself burned out and had to be replaced four times during our study. Moreover, the performance of the electric on-demand heaters was sensitive to the influent temperatures. For example, when the influent was below 12 °C, as can be the case during winter months, the target temperature could not be achieved. Other studies have reported similar issues in the reliability and temperature control of electric on-demand systems in delivering hot water to the POU.^27^

### Challenges of Sustaining Legionella Growth in Experimental Systems

During the first 47 months of the experiment, sustainable levels of *L. pneumophila* could not be maintained. This unfortunately detracted from the ability to then test the effects of the various heater configurations on *L. pneumophila* but also reflects the reality that *L. pneumophila* is fastidious and the exact combinations of conditions (water temperatures, nutrient conditions, plumbing materials, etc.) that consistently support that its growth under real-world drinking water conditions are not fully understood.^28,29^ This is a cautionary tale for other studies seeking to expand knowledge of the behavior of *L. pneumophila* in situ. Successful growth of *L. pneumophila* was eventually achieved after 48–66 months of operation using this apparatus, which we hypothesize is due to the need for mature biofilms to establish. Other studies have indicated that 3+ years can be required for a mature drinking water biofilm to fully form.^30^ Thus, in this study, we focus on spatial and temporal patterns in temperature stratification throughout the systems and their correspondence to *Legionella* and *L. pneumophila* growth ranges reported in the literature.

### Effect of Temperature Settings and Recirculation on Suitable for *Legionella* Growth

At 48 °C, the recirculating tank maintained temperatures much more suitable for *Legionella* growth than the standard tank. However, increasing set points >60 °C reverses the relative risk of standard versus recirculating tanks, since the recirculating volume would be held above 55 °C at the higher temperature set point throughout the tank (Figure 5). Even with a set point of 66 °C, the standard configuration will continue to maintain portions of the tank in a temperature range at risk for *Legionella* growth (Figures 5 and 6). Stratification can have a large impact on the microbial ecology in premise plumbing, with *L. pneumophila* found at the highest concentrations and associated with a reduction in temperature at the bottom of electric water heaters.^31^ The low 40 °C target in phase 1 and the high 66 °C target in phase 2 were selected to capture a range well below those recommended for *Legionella* control, up to a range of scalding concern.^21^ These temperatures also fall within those encountered in household water heaters in the field, according to a prior study of flushed heaters in Flint, MI, reporting temperatures of 33–67 °C.^13^

While the use of recirculating systems is more common in large commercial buildings than in residential homes,^31^ the 2022 California Green Building Standards Code recommends that one- and two-family dwellings have recirculation systems for hot water.^32^ There are many different types of recirculation systems and plumbing system designs, for both residential and commercial applications, which may not approach the complete mix ideal achieved herein.

### Insulation and Heater Elements Influence the Volume of Standard Tanks at Risk of *L. pneumophila* Growth

Phase 2 illustrated how the addition of an *R*-20 insulating floor pad can serve as a powerful and economical approach to limiting the *Legionella* growth risk in standard tanks. Use of such a pad at a set point of 66 °C dramatically reduced the tank volume at risk for *Legionella* growth. However, at a set point of 52 °C and below, the added insulation warmed the cold water at the very bottom of the tank and actually increased the volume at risk for *Legionella* growth. Also, a lower heating element operating at a cooler temperature, or burned out entirely, could have substantial effects on the volume of a conventional tank at risk for *Legionella* growth and could potentially go undetected by the consumer because the pattern in hot water delivered to the tap is not as strongly affected.

Older water heaters often have less insulation than modern heaters.^33^ It is also reasonable to expect that the temperature of the floor on which the water heater sits is an important factor governing the tank temperature. The insulation experiments herein were conducted on the first floor of a well-insulated building constructed in 2009, where the temperature of the concrete floor was measured at 19.4 °C. Many floors on which water heaters are still likely much colder, especially in the winter. For example, a cold basement floor in a northern climate^34^ would be expected to cool the bottom of a tank to a greater extent than what was observed in the present study, and the floor insulation would have much more dramatic impacts. In contrast, a field study conducted in the hot climate of Florida indicated that buildings with recirculating systems had higher risks of *Legionella* colonization than buildings that did not when the water utility utilized chlorine as its primary disinfectant.^35^ Florida’s warm climate, in tandem with the temperature each building’s hot water was set to, could have led to the recirculating systems in each building distributing water temperatures capable of *Legionella* colonization throughout the buildings with recirculating systems that were sampled. Clearly, local factors such as climate, room temperature, and the temperature of the floor under the water heater, as well as insulation status, may influence temperature profiles in water heaters and, as a result, impact the growth potential of *Legionella* and other opportunistic pathogens.

Insulation pads can also prevent condensation and corrosion by lifting the tank off a moist floor, increasing the tank lifetime and also saving energy. For conventional electric water heaters that experience significant heat loss,^24^ the use of insulation can reduce operating costs, while also constraining available *Legionella’s* ecological niche within the heater.

## Conclusion

- In a controlled, head-to-head study at 48 °C, recirculating tank water systems had greater temperature variability in delivered water than a standard tank system.
- The most energy efficient system was the on-demand (tankless) system, but the electric versions of these heaters had limitations that can reduce user comfort. The standard tank had less tank volume (26%) within the very high-risk range for *Legionella* growth compared to the recirculating tank (94%) at a set point of 48 °C. However, the relative risk of the standard tank was higher if the set point was 66 °C. These findings can help explain why recirculating systems are sometimes at higher risk of *Legionella* growth compared to standard systems, and vice versa.
- The very low storage volume and lower surface area of the tankless system plus distal pipes resulted in relatively trivial volumes of water at risk for *Legionella* growth.
- Use of insulation, lower heater element set point, lower heater element status, can dramatically alter the temperature profile throughout a conventional tank system, and dependent on circumstance could either increase or decrease the volume at risk for *Legionella* growth. Judicious use of insulation can holistically help alter the public health risks of *Legionella* while saving energy.

## Supporting Information Available

Information regarding temporal patterns, influent temperature, system capacity, power demand, surface area to volume ratios, and internal tank temperature sampling apparatus used for the configurations in phase 1 can be found in the attached Supporting Information document; additionally, the time needed for the water heater used in phase 2 to stabilize can also be found in the attached supplemental document (PDF)
