# Supplementary material for: Water Heater Type, Temperature Setting, Operational Conditions, and Insulation Affect Ecological Niches for Legionella Growth
Source: ACS ES T Water. 2024 Dec 23;5(1):377–86. doi: 10.1021/acsestwater.4c00894 (PMC11731276; doi:10.1021/acsestwater.4c00894)
Supplement: Supplementary file 1 — ew4c00894_si_001.pdf [file ew4c00894_si_001.pdf]

Supporting Information

for

Water Heater Type, Temperature Setting, Operational Conditions, and Insulation Affect Ecological Niches for *Legionella* Growth

Authors:

Fernando A. Roman Jr*‡[1], Rebekah L. Martin‡[2], William J. Rhoads[3], Annie Pearce[4], Rania E. Smeltz[1], Amy Pruden[1], Marc A. Edwards[1]

[1]Department of Civil and Environmental Engineering, Virginia Tech, Blacksburg, Virginia 24061, United States

[2]Department of Civil and Environmental Engineering, Virginia Military Institute, Lexington, Virginia, 24450, United States

[3]Black and Veatch Corporation, Overland Park, Kansas, 66211, United States

[4]Department of Building Construction, Virginia Tech, Blacksburg, Virginia 24061, United States

‡These two authors are designated as co-first authors and contributed equally to this work

*Corresponding Author froman@vt.edu

Contents:

Supplementary Table 1: Sample information for temporal patterns and influent temperature

Supplementary Table 2: Power, capacity, and surface area to volume ratios of three water heater configurations utilized in Phase 1

Supplementary Figure 1: Internal tank temperature sampling rod apparatus used in Phase 1

Supplementary Figure 2: Time required for Water heater used in Phase 2 to stabilize

Supplementary Table 1. Sample information for energy efficiency calculations. Sample numbers vary because the data reported only corresponds to sampling days where influent temperature measurements were taken alongside energy consumption for one flush. Monitoring period lengths varied from eight weeks to two weeks for each flush rate and temperature setting combination.

**Flush Rate**	**Set Temp (°C)**	**Season†**	**Influent Temp (°C)**	**n**
Low	40	Summer/Winter	18.4 ± 5.4	5
	48	Fall	20.6 ± 0.8	4
Medium	40	Spring	12.3 ± 0.2	4
	48	Fall	20.0 ± 1.4	2
High	40	Spring	14.9 ± 1.8	4
	48	Winter/Spring	16.7 ± 1.5	3

†Summer = June – August, Fall = September – November, Winter = December – February, Spring = March – May

Supplementary Table 2: Capacities, surface area to volume ratios, and power demands for each water heater configuration tested in Phase 1.

**Configuration**	**System capacity (liters)**		**Surface area of the whole system (m^2)**	**Surface area to volume ratio of the whole system (m^{-1})**	**Surface area to volume ratio of each heater (m^{-1})**	**Surface area to volume ratio of the pipes (m^{-1})**	**Power demand of each heater (Watts)**
	**Tank/ Heater**	**Pipes**					
Standard	151	4.3	2.68	16.5	9.6	209	4500
Recirculating	151	8.7	3.59	21.6	9.6	209	4500
On-Demand	0.11	4.6	1.03	213	314	209	9500

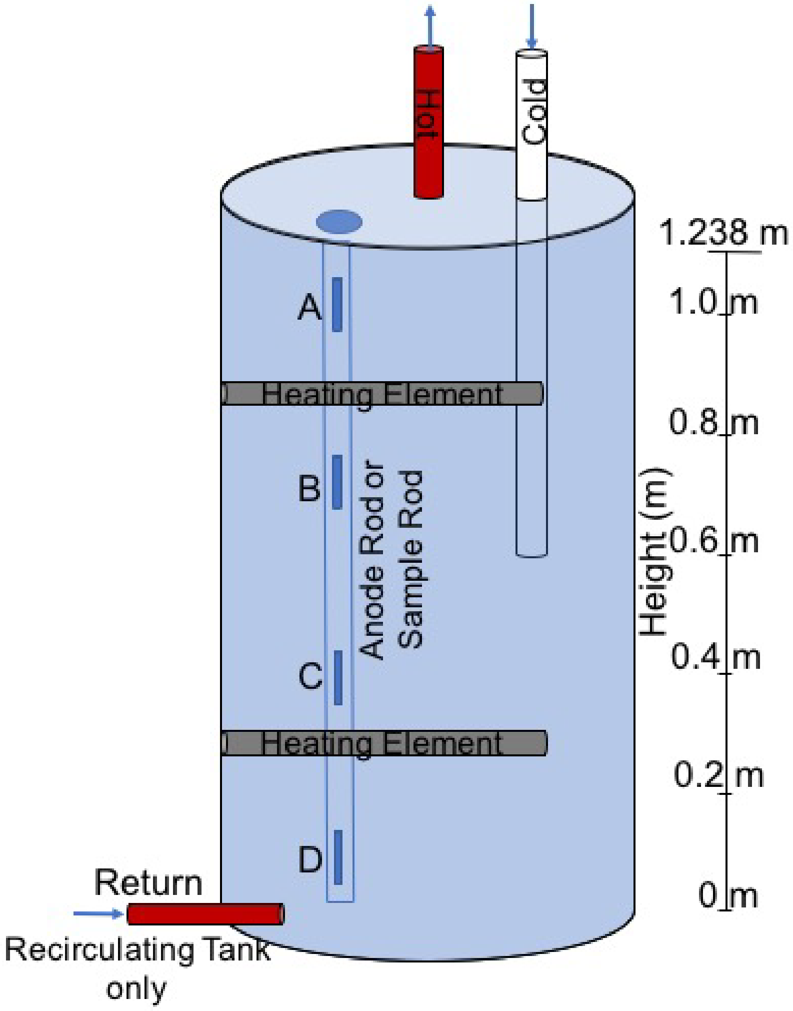

Supplementary Figure 1: Sampling method for internal temperature measurements of tank water heaters. The anode rod was switched out with the sample rod for the duration of time needed to take the measurements. Four wireless data logger temperature probes (HOBO model U12) were attached to the sampling rod to record temperature inside of the tank.

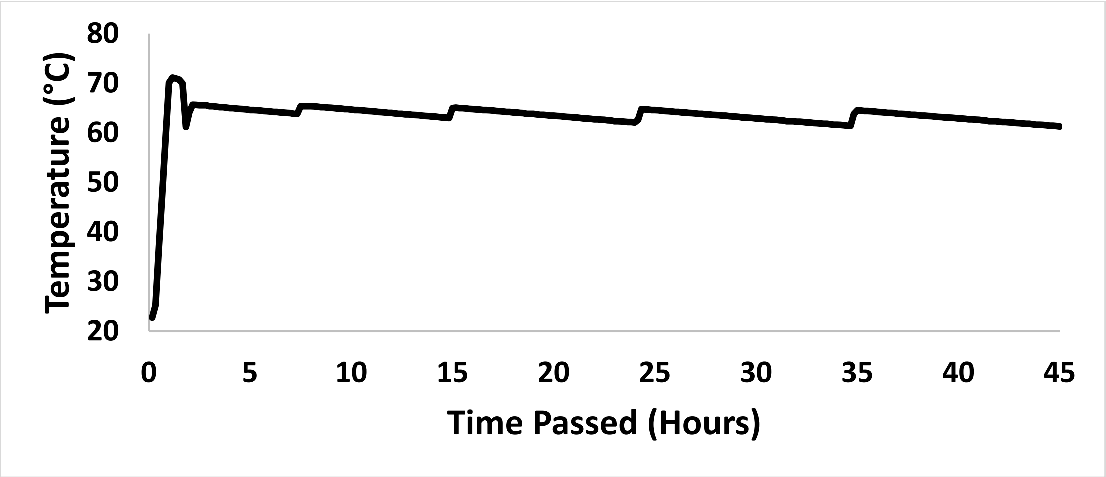

Supplementary Figure 2. Temperature of water near the top area of the conventional water heater (data logger placed 7.6 cm below anode rod hole) used for insulation experiments, measured over 45 hours. Temperature logged in condition where the water heater had no added insulation, demonstrating that 45 hours was sufficient to achieve a steady state. It was shown that the temperature of the bulk water in the top area of the water heater stabilized condition after just two hours. The cycling of temperature up and down (61.1°C – 65.6°C) thereafter is due to the normal on/off thermostatic controlled heating and cooling cycle.
